# Nonhuman TRIM5 Variants Enhance Recognition of HIV-1-Infected Cells by CD8^+^ T Cells

**DOI:** 10.1128/JVI.00819-16

**Published:** 2016-09-12

**Authors:** Esther Jimenez-Moyano, Alba Ruiz, Henrik N. Kløverpris, Maria T. Rodriguez-Plata, Ruth Peña, Caroline Blondeau, David L. Selwood, Nuria Izquierdo-Useros, Arnaud Moris, Bonaventura Clotet, Philip Goulder, Greg J. Towers, Julia G. Prado

**Affiliations:** a AIDS Research Institute, IrsiCaixa, Hospital Germans Trias i Pujol, Badalona, Spain; b KwaZulu-Natal Research Institute for TB and HIV, University of KwaZulu-Natal, Durban, South Africa; c Division of Infection and Immunity, University College London, London, United Kingdom; d The Wolfson Institute for Biomedical Research, University College London, United Kingdom; e Sorbonne Universités, UPMC University Paris 6, INSERM U1135, CNRS ERL 8255, Center for Immunology and Microbial Infections—Paris, Paris, France; f Department of Paediatrics, University of Oxford, Oxford, United Kingdom; Emory University

## Abstract

Tripartite motif-containing protein 5 (TRIM5) restricts human immunodeficiency virus type 1 (HIV-1) in a species-specific manner by uncoating viral particles while activating early innate responses. Although the contribution of TRIM5 proteins to cellular immunity has not yet been studied, their interactions with the incoming viral capsid and the cellular proteasome led us to hypothesize a role for them. Here, we investigate whether the expression of two nonhuman TRIM5 orthologs, rhesus TRIM5α (RhT5) and TRIM-cyclophilin A (TCyp), both of which are potent restrictors of HIV-1, could enhance immune recognition of infected cells by CD8^+^ T cells. We illustrate how TRIM5 restriction improves CD8^+^ T-cell-mediated HIV-1 inhibition. Moreover, when TRIM5 activity was blocked by the nonimmunosuppressive analog of cyclosporine (CsA), sarcosine-3(4-methylbenzoate)–CsA (SmBz-CsA), we found a significant reduction in CD107a/MIP-1β expression in HIV-1-specific CD8^+^ T cells. This finding underscores the direct link between TRIM5 restriction and activation of CD8^+^ T-cell responses. Interestingly, cells expressing RhT5 induced stronger CD8^+^ T-cell responses through the specific recognition of the HIV-1 capsid by the immune system. The underlying mechanism of this process may involve TRIM5-specific capsid recruitment to cellular proteasomes and increase peptide availability for loading and presentation of HLA class I antigens. In summary, we identified a novel function for nonhuman TRIM5 variants in cellular immunity. We hypothesize that TRIM5 can couple innate viral sensing and CD8^+^ T-cell activation to increase species barriers against retrovirus infection.

**IMPORTANCE** New therapeutics to tackle HIV-1 infection should aim to combine rapid innate viral sensing and cellular immune recognition. Such strategies could prevent seeding of the viral reservoir and the immune damage that occurs during acute infection. The nonhuman TRIM5 variants, rhesus TRIM5α (RhT5) and TRIM-cyclophilin A (TCyp), are attractive candidates owing to their potency in sensing HIV-1 and blocking its activity. Here, we show that expression of RhT5 and TCyp in HIV-1-infected cells improves CD8^+^ T-cell-mediated inhibition through the direct activation of HIV-1-specific CD8^+^ T-cell responses. We found that the potency in CD8^+^ activation was stronger for RhT5 variants and capsid-specific CD8^+^ T cells in a mechanism that relies on TRIM5-dependent particle recruitment to cellular proteasomes. This novel mechanism couples innate viral sensing with cellular immunity in a single protein and could be exploited to develop innovative therapeutics for control of HIV-1 infection.

## INTRODUCTION

Early immunity against viral infections is critical in controlling disease course (1). In the case of HIV-1, early immunity is thought to be too late and too weak to control the irreversible damage established during acute infection through viral cytopathic effects (2). A combination of potent early innate and adaptive immune responses is required for effective virological control and sustained protection against viral infections (3).

Innate antiviral proteins, also called restriction factors, are the first intracellular barriers against HIV-1 infection. Restriction factors mediate rapid viral sensing, thus enabling HIV-1 suppression within hours and before adaptive immunity can be engaged. Tripartite motif-containing protein 5 (TRIM5) exhibits one of the strongest signatures of evolutionary selection pressure in mammalian genomes and mediates cross-species recognition of retroviruses (4, 5). TRIM5 variants from Old World monkeys, such as rhesus macaques, restrict a broad spectrum of human retroviruses (HIV-1 and HIV-2) and animal retroviruses (equine infectious anemia virus [EIAV] and N-tropic murine leukemia virus [N-MLV]). Meanwhile, New World monkeys do not generally restrict HIV-1 (6). An exception is found in New World owl monkeys, where TRIM5 has gained a cyclophilin A-derived virus-binding domain and restricts HIV-1 very efficiently.

Although the precise molecular interactions between HIV-1 and TRIM5 are not fully understood, TRIM5 has two complementary antiviral functions that both rely on the recognition of the HIV-1 capsid lattice. The first is its function as a restriction factor, through direct binding to the incoming retrovirus and disruption of the capsid by a proteasome-dependent TRIM5 mechanism (7–9). The second is its function as a pattern recognition receptor, which it carries out by promoting the secretion of type I interferons (IFNs) (10). Thus, innate cellular recognition by TRIM5 constitutes a host frontline defense against initial viral spread.

Together with innate viral sensing, cellular immune responses, and particularly HIV-1-specific CD8^+^ T-cell responses, are crucial for the control of both acute and chronic viral infections. The key role of adaptive immunity in control of HIV-1 infection is seen clearly in the associations between the expression of specific HLA class I molecules and HIV-1 disease outcome (11–13), the breadth of Gag-specific CD8^+^ T-cell responses and virological control (14, 15), and the emergence of immune escape variants against CD8^+^ T-cell responses (16, 17). Moreover, recent studies have demonstrated how some restriction factors (APOBEC3G and SAMHD1) can modify cellular immunogenicity and recognition of HIV-1-infected cells by CD8^+^ T cells (18, 19), thus suggesting a complex interdependency between intracellular innate viral sensing and adaptive immunity.

In the case of TRIM5, the interaction with the adaptive immune system remains unknown. However, knowledge of such an interaction might be crucial for the development of novel therapeutic strategies, which would seek to combine potent intracellular viral sensing with antiviral CD8^+^ T-cell responses to generate protective immunity against HIV-1. Here, we describe a series of experiments to underscore the role of TRIM5 in CD8^+^ T-cell antiviral activity. We studied TRIM5 variants that potently restrict HIV-1 infection (rhesus TRIM5α [RhT5] and New World owl monkey TRIM-cyclophilin A [TCyp]) and developed an *in vitro* model of RhT5 and TCyp expression in HIV-1-susceptible cell lines. Using this model, we evaluated HIV-1 restriction and changes in the antigenicity of infected cells for antiviral CD8^+^ T-cell recognition. Furthermore, we tested the specific contribution of TRIM5 variants to the activation of HIV-1-specific CD8^+^ T cells and evaluated whether the resulting interactions depend on CD8^+^ T-cell protein specificity. Our data demonstrate the direct contribution of TRIM5 variants to induction of HIV-1-specific CD8^+^ T-cell responses by coupling innate and adaptive immunity in HIV-1-infected cells.

## MATERIALS AND METHODS

### Generation of stable U937 cell lines expressing nonhuman TRIM5 variants.

In order to assess epitope presentation in the context of nonhuman TRIM5 expression, we used a U937 cell line that was transfected and selected for the expression of HLA-B*2705 (20). The U937 HLA-B*2705 cells were transduced with lentiviral vectors that confer puromycin resistance and encode either of the two TRIM5 isoforms, namely, the rhesus TRIM5α (RhT5) or the owl monkey TRIM cyclophilin A (TCyp) fusion gene. Control cells were transduced with the empty lentiviral vector. Transduced cell lines were selected in the presence of 1.25 μg/ml of puromycin (Sigma) and maintained in medium containing RPMI 1640 medium (Invitrogen), 10% fetal calf serum, penicillin (100 U/ml), streptomycin (100 μg/ml), G418 (0.5 mg/ml), and puromycin (1.25 μg/ml) (Sigma). We quantified TRIM5 mRNA in transduced cells. Briefly, RNA was extracted from 5 × 10^6^ U937 cells expressing an empty vector (EV), RhT5, or TCyp (RNeasy RNA extraction kit; QIAgen, United Kingdom). The RNA extracted was treated with DNase (Turbo DNA-free kit; Thermo Fisher Scientific) to remove residual DNA. cDNA from 1 μg of DNase-treated RNA was synthesized with SuperScript III reverse transcriptase (ThermoFisher Scientific). cDNA was diluted 1:5 in nuclease-free H_2_O, and 2 μl was used for real-time PCR quantification with SYBR green PCR master mix (Applied Biosystems) in a 7500 Fast Real-Time PCR system (Applied Biosystems) and in three technical triplicates with the following primers: RhT5 forward (FWD), 5′-CGCTACTGGGTTGATGTGACAC-3′, and RhT5 reverse (REV), 5′-CCCTGGTGCCTGATACATTATCTG-3′ (21); TCyp FWD, 5′-CAGAAGTCCAACGCTACTGGG-3′, and TCyp REV, 5′-CTTGCCACCAGTGCCATTATGG-3′ (22). RhT5- and TCyp-coding plasmids were used as standards for quantification of mRNA in each gene.

### Virus and CD8^+^ T-cell lines.

Virus HIV-1_GFP_ was produced by cotransfecting the HIV-1 packaging plasmid pCMVR8.2, the green fluorescent protein (GFP)-encoding vector genome pCSGW, and the vesicular stomatitis virus (VSV) G protein-encoding plasmid pMDG in a ratio of 4:1:1 into 293T cells using FuGENE. Viruses were harvested at 48 h posttransfection. Viral stocks were purified through a 20% sucrose cushion, concentrated by ultracentrifugation at 4°C and 24,000 rpm for 90 min, and stored at −80°C. The 50% tissue culture infective dose (TCID_50_) for HIV-1_GFP_ particles was determined in EV-expressing U937 cells by limiting dilution for 48 h postinfection using the Reed and Muench method. The NL43-VprGFP virus was produced by calcium phosphate cotransfection of a plasmid expressing NL4-3 and one expressing Vpr-GFP at a 1:1 ratio (23) into 293T cells. Viruses were harvested at 48 h posttransfection, purified, and concentrated as previously mentioned.

The HLA-B*2705-restricted CD8^+^ T-cell lines specific for the HIV-1 KK10 epitope in Gag and the KY9 epitope in Pol and the HLA-B*5701-restricted CD8^+^ T-cell lines specific for the HIV-1 KF11 epitope were obtained as previously described (24). Cells were cultured in H10 medium containing RPMI 1640 medium (Invitrogen) supplemented with 10% AB human serum (Invitrogen), 10% natural T-cell grow factor (TCGF) (Helvetica Healthcare), 100 U/ml of penicillin, and 100 μg/ml of streptomycin (Invitrogen). After 3 days in culture, cells were fed with H10 medium, and once a week the cultures were stimulated with a mixture of 1:1 irradiated peptide-pulsed (10 μg/ml) autologous B-cell lines and peripheral blood mononuclear cells (PBMCs) from three HIV-1-seronegative donors.

### HIV-1 restriction experiments.

U937 cells expressing RhT5, TCyp, and the control (EV) were infected with a VSV-pseudotyped HIV-1_GFP_ at a dose equivalent to a nominal multiplicity of infection (MOI) of 0.2 on EV-expressing cells for 2 h at 37°C. In some experiments, cyclosporine (CsA; 5 μM) or sarcosine-3(4-methylbenzoate)–CsA (SmBz-CsA; 5 μM) was added to the culture at the time of infection. GFP was measured by flow cytometry at 22 h postinfection.

### CD8^+^ T-cell inhibition and killing experiments.

The EV-, RhT5-, and TCyp-expressing cells were infected with HIV-1_GFP_ in the presence of 5 μM CsA at a dose equivalent to an MOI of 0.2 on EV-expressing cells. After 2 h of infection, cells were cultured in the absence or the presence of HLA class I-matched CD8^+^ T cells at an effector-to-target ratio (E/T) of 1:2 (24). Moreover, to control for unspecific CD8^+^ T-cell activation, HIV-1-infected cells were cultured in the presence of HLA class I-mismatched (MM) CD8^+^ T cells. Approximately one-third of the culture was collected at 3, 6, and 22 h postinfection, and Live/Dead staining and staining for expression of CD4, CD8, and GFP in infected cells were carried out for all samples. HIV-1 CD8^+^ T-cell inhibition was calculated as follows: 100 − [(% GFP^+^ cells in CD4^+^ coculture with CD8^+^ cells/% GFP^+^ cells in CD4^+^ cells in the absence of CD8^+^) × 100] (where GFP^+^ is GFP-positive). The number of live CD4^+^ cells was calculated as follows: [% live CD4^+^ cells in coculture with CD8^+^ cells/% live CD4^+^ cells in the absence of CD8^+^ cells] × 100. In addition, to verify the functionality of the CD8^+^ T cells used in the experiments, we measured the frequency of MIP-1β/CD107a-positive cells in response to CD8^+^ T-cell coculture with HLA class I-matched B-cell lines loaded with cognate peptide.

### CD8^+^ T-cell functionality.

HLA class I-matched B-cell lines and EV-, RhT5-, and TCyp-expressing cells were loaded with cognate peptide (2 μg/ml) for 1 h in the presence of cyclosporine (5 μM), SmBz-CsA (5 μM), or no drugs. After that, CD8^+^ T cells were added to the culture in an E/T ratio of 2:1 in the presence of CD107a and incubated for 3 h in the presence of brefeldin A (10 μg/ml) and GolgiStop. CD8^+^ T-cell functionality was examined by measuring the proportion of CD107a/MIP-1β-positive cells by intracellular cytokine staining.

### CD8^+^ T-cell activation experiments. (i) Flow cytometry.

RhT5- and TCyp-expressing cells were infected with HIV-1_GFP_ at an MOI of 0.2 in the presence of aztreonam (AZT; 5 μM), CsA (5 μM), or SmBz-CsA (5 μM) or without drugs. After 2 h of infection, Gag or Pol HLA class I-matched CD8^+^ T cells were added to the culture in the presence of CD107a at an E/T ratio of 1:2. In addition, HLA class I-mismatched CD8^+^ T cells were included to control for nonspecific activation. Samples were collected at 22 h postinfection and stained with Live/Dead stain and for CD4, and CD8 surface markers and CD107a and MIP-1β intracellular expression. To compare experiments, the frequency of CD8^+^ T-cell activation was calculated as relative activation in relation to the maximum level of CD107a/MIP-1β expression achieved in the experiment for any of the conditions tested as 100%.

### (ii) IFN-γ ELISPOT.

RhT5- and TCyp-expressing cells were infected with HIV-1_GFP_ at a dose equivalent to an MOI of 0.5 on EV-expressing cells for 24 h. Infected cells were cocultured with HIV-1 Gag- or Pol-specific CD8^+^ T cells at various E/T ratios (1:16, 1:8, 1:4, and 1:2) and incubated overnight. For positive controls, RhT5- and TCyp-expressing cells were loaded with cognate peptide and cocultured with HIV-1 Gag- or Pol-specific CD8^+^ T cells. For negative controls, RhT5- and TCyp-expressing target cells alone were cocultured with HIV-1 Gag- or Pol-specific CD8^+^ T cells. IFN-γ production was measured using enzyme-linked immunosorbent spot (ELISPOT) assays, and the background production by negative controls was subtracted from all samples.

In order to test additional HIV-1-specific CD8^+^ T cells, H9-B*5701 cells were transiently transduced with the EV (empty vector)-expressing and rhesus TRIM5α (RhT5)-expressing lentiviral vectors that confer puromycin resistance. After 72 h of lentiviral transduction and 48 h in puromycin (1.25 μg/ml) (Sigma) cells were infected with HIV-1_GFP_ at an MOI of 0.5 for 24 h. Infected cells were cocultured with HLA-class I-matched HIV-1 Gag-specific CD8^+^ T cells at an E/T ratio of 1: 2 overnight. H9 EV- and RhT5-expressing cells were loaded with cognate peptide and cocultured with HIV-1 Gag-specific CD8^+^ T cells for positive controls. We performed a similar experiment in U937 HLA-B*2705 cells in cocultures with HLA-class I-matched HIV-1 Gag-specific CD8^+^ T cells as the control.

### Surface and intracellular antibody staining.

Depending on the experiments, cell populations were labeled with combinations of the following antibodies: Live/Dead stain (APC-CY7; Invitrogen), CD4 (clone SK3; BD), CD8 (clone RPA-T8; BD), p24 (clone KC57-RD1; Coulter Clone), MIP-1β (clone 24006; R&D Systems), and CD107a (clone H4A3; BD). The intracellular staining assay was performed as described previously (25), with some modifications. Briefly, CD107a was added to the culture with CD8^+^ T cells and incubated for an additional 3 h in the presence of brefeldin A (10 μg/ml) and GolgiStop. Cells were then stained first with Live/Dead stain and for surface markers of CD4 and CD8 and fixed with 1% formaldehyde overnight. The following day, the cells were permeabilized with buffer containing saponin and stained for p24 and/or MIP-1β. All samples were fixed for 2 h with 1% formaldehyde before being acquired on an LSRII flow cytometer. Data were analyzed with FlowJoV (Tree Star Inc.).

### Immunofluorescent staining.

U937 cells expressing EV, RhT5, and TCyp or HeLa cells expressing EV, RhT5, or TCyp were infected with NL43-VprGFP for 2 h at 37°C. In addition, EV- and TCyp-expressing cells in the absence or presence of 5 μM SmBz-CsA were infected with NL43-VprGFP for 2 h at 37°C. For the different experiments, following infection cells were fixed, stained for the 19S proteasome subunit (BML-PW8825; Enzo Life Sciences), and revealed with the secondary antibody Alexa Fluor 555 (A 21424; Invitrogen). Cells were cytospun onto coverslips and mounted with 4′,6′-diamidino-2-phenylindole (DAPI) mounting medium (Invitrogen). Images were collected as described by Izquierdo-Useros et al. (26) with an Ultraview ERS spinning disk system (Perkin-Elmer) mounted on a Zeiss Axiovert 200 M inverted microscope. To obtain three-dimensional (3-D) reconstructions and count virus/proteasome contacts, confocal *Z* stacks were processed with Volocity software (Perkin-Elmer) using the isosurface module.

### Statistical analyses.

Statistical tests were performed using GraphPad Prism (GraphPad Software, Inc.). *P* values were calculated using a Mann-Whitney test, Wilcoxon signed-rank test, or two-way analysis of variance (ANOVA). Spearman correlation coefficients were calculated between p24 and GFP expression. *P* values of <0.05 were considered statistically significant.

## RESULTS

### Nonhuman TRIM5 variants improve inhibition of HIV-1-infected cells by CD8^+^ T cells.

**FIG 1:**
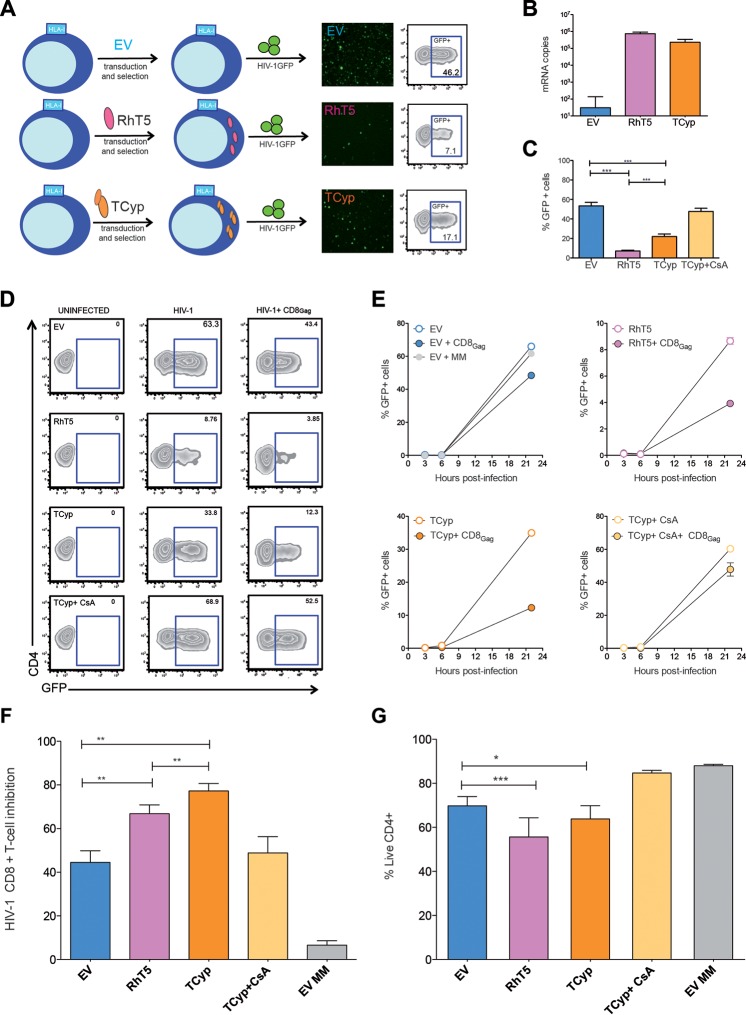
HIV-1-specific CD8^+^ T-cell inhibition in HIV-1-infected cells expressing TRIM5. (A) U937 cells were transduced with a lentiviral empty vector (EV) or vectors coding for rhesus TRIM5α (RhT5) or owl monkey TRIM-cyclophilin A (TCyp) and conferring resistance to puromycin. Stable transduced cell lines with EV, RhT5, and TCyp expression were infected with vesicular stomatitis virus (VSV)-pseudotyped HIV-1 GFP (HIV-1_GFP_), and retroviral restriction was quantified according to the frequency of GFP^+^ cells by flow cytometry. (B) mRNA copy numbers of TRIM5 in U937 cells expressing EV, RhT5, and TCyp. The graph shows the mean ± standard error of the mean of three technical replicates. (C) Percentage of GFP^+^ cells in cells expressing EV, RhT5, TCyp, and TCyp in the presence of 5 μM CsA after infection with HIV-1_GFP_. The graph shows the mean ± standard error of the mean of six independent experiments performed in triplicate. The *P* values were calculated using a Mann-Whitney test. (D) Zebra plots representative of HIV-1-specific CD8^+^ T-cell-mediated inhibition in infected cells expressing EV, RhT5, TCyp, and TCyp plus CsA. The percentage of GFP^+^ cells at 22 h postinfection was determined in uninfected HIV-1-infected cells and in HIV-1-infected cells in coculture with HLA class I-matched Gag-specific CD8^+^ T cells. (E) Kinetics of HIV-1 infection in the absence (empty dots) or presence (colored dots) of HLA class I-matched CD8^+^ T cells in cells expressing EV, RhT5, TCyp, and TCyp plus CsA, as indicated. EV+ MM, cells expressing EV in the presence of HLA class I-mismatched CD8^+^ T cells. (F) HIV-1-specific CD8^+^ T-cell-mediated inhibition in infected cells expressing EV, RhT5, TCyp, and TCyp plus CsA at 22 h postinfection. EV MM, EV-expressing cells in coculture with HLA class I-mismatched CD8^+^ T cells. The graph shows the mean ± standard error of the mean of three independent experiments performed in triplicate. The *P* values were calculated using a Wilcoxon signed-rank test. (G) Frequency of live CD4-positive cells in EV-, RhT5-, TCyp-, and TCyp-CsA-expressing cells infected with HIV-1 in coculture with CD8^+^ T cells. The graph shows the ratio of live cells in the presence and absence of CD8^+^ T cells. The bars represent the mean ± standard error of the mean of three independent experiments performed in triplicate. The *P* values were calculated using two-way ANOVA corrected by the Bonferroni method for multiple comparisons. Only significant values are shown in the figures (***, *P* < 0.0001; **, *P* < 0.001; *, *P* < 0.01). Experiments shown in panels C to G were performed at an MOI of 0.2.

In order to investigate the role of TRIM5 in retroviral restriction and CD8^+^ T-cell antiviral activity, we expressed two nonhuman TRIM5 orthologs (RhT5 and TCyp) that potently restrict HIV-1 using lentiviral vectors in U937 cells. The same lentiviral EV was used as a control (Fig. 1A). Since U397 cells are susceptible to HIV-1 infection and express HLA-B*2705, we can rapidly evaluate HIV-1 infectivity and CD8^+^ T-cell inhibition (20, 24). In addition, the VSV-pseudotyped HIV-1 particles that express GFP upon viral integration (HIV-1_GFP_) used here enabled us to easily measure TRIM5 antiviral activity (Fig. 1A). TRIM5 mRNA expression levels were similar between transduced cell lines (Fig. 1B). As shown in Fig. 1C, RhT5- and TCyp-expressing cells restricted HIV-1 significantly more than EV-expressing control cells (RhT5, 6.98%; TCyp, 19.17%; EV, 53.8%; mean values, *P* < 0.0001 for all data set comparisons) (Fig. 1C). Moreover, the restriction phenotype was consistent at different MOIs (0.2 and 0.5) (data not shown) and specific to TRIM5 expression, as observed by blocking TCyp activity with CsA, which binds to the cyclophilin domain in TCyp and fully rescues HIV-1 infectivity (Fig. 1C).

We next assessed whether TRIM5 expression in HIV-1-infected cells affects suppression mediated by HIV-1-specific CD8^+^ T cells. We infected EV-, RhT5-, and TCyp-expressing cells with HIV-1_GFP_ and cocultured them with HLA class I-matched HIV-1 Gag-specific CD8^+^ T cells. To control for nonspecific CD8^+^ T-cell activation, we cocultured infected cells with HLA class I-mismatched (MM) CD8^+^ T cells under similar conditions. HIV-1 CD8^+^ T cell-mediated inhibition was assessed by differences in GFP expression levels over time (3, 6, and 22 h postinfection) in the presence or absence of CD8^+^ T cells (Fig. 1D and E). We consistently demonstrated significant increases in HIV-1-specific, CD8^+^ T-cell-mediated inhibition of HIV-1-infected cells expressing TCyp (77.2%) and RhT5 (66.8%), compared with control EV-expressing cells (44.5%) (TCyp versus EV, *P* = 0.003; RhT5 versus EV, *P* = 0.003). The strongest effect was observed with TCyp (Fig. 1F) (TCyp versus RhT5, *P* = 0.003). Critically, this effect reverted to background levels when TCyp activity was blocked using CsA.

Moreover, differences in HIV-1-specific CD8^+^ T-cell inhibition levels translate as a reduction of live RhT5- and TCyp-expressing cells infected with HIV-1 in the presence of CD8^+^ cells (TCyp versus EV, *P* = 0.0007; RhT5 versus EV, *P* = 0.013) (Fig. 1G). TCyp had the strongest effect on CD8^+^ T-cell inhibition but not on total killing of infected cells (Fig. 1F and G). This discrepancy could be due to the fact that the weaker restriction mediated by TCyp leaves more infected T cells to be inhibited by CD8^+^ T cells despite the absence of differences in total cell deaths between RhT5- and TCyp-expressing cells. Nevertheless, our data demonstrate that both RhT5 and TCyp restriction factors increase the antigenicity of HIV-1-infected cells and improve HIV-1-specific CD8^+^ T-cell killing of infected cells.

### TRIM5 expression in HIV-1-infected cells enhances activation of HIV-1-specific CD8^+^ T cells.

**FIG 2:**
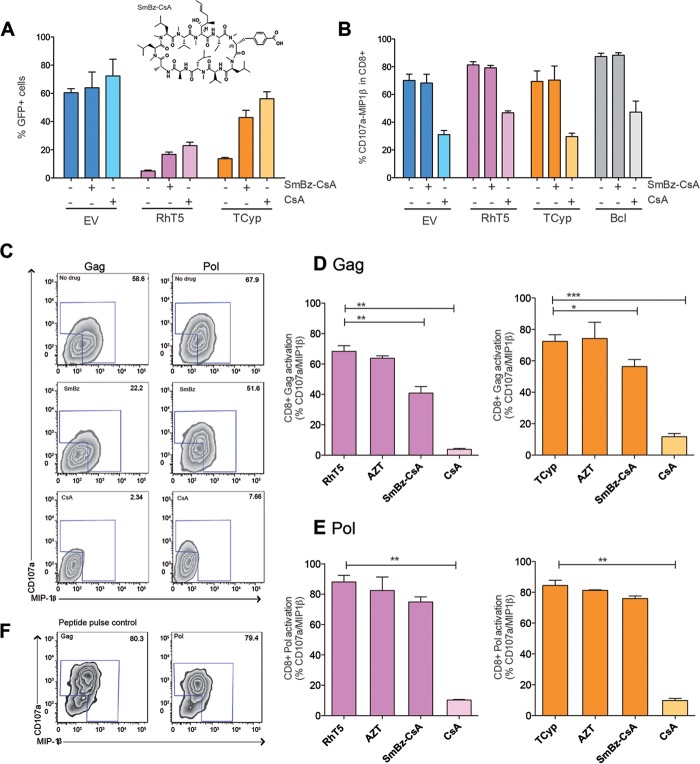
HIV-1-infected cells expressing TRIM5 variants enhance CD8^+^ T-cell activation. (A) Frequency of GFP^+^ cells in EV-, RhT5-, and TCyp-expressing cells in the presence or absence of 5 μM SmBz-CsA or CsA. The SmBz-CsA molecule is shown above the graph. Data are shown as the mean ± standard error of the mean from three independent experiments performed in triplicate. (B) Frequency of CD107a/MIP-1β expression in HIV-1-specific CD8^+^ T cells cocultured in response to EV, RhT5, and TCyp and in a B-cell line (BCl) loaded with cognate peptide. The data are from two independent experiments performed in duplicate. (C) Representative zebra plot of the frequency of CD107a/MIP-1β expression in HIV-1 Gag- or Pol-specific CD8^+^ T cells in coculture with infected cells expressing RhT5 in the presence or absence of SmBz-CsA or CsA. (D) Activation levels were measured by the frequency of CD107a/MIP-1β in HIV-1 Gag-specific CD8^+^ T cells cocultured with RhT5- or TCyp-expressing cells infected with HIV-1 in the presence or absence of AZT, SmBz-CsA, or CsA. (E) Activation levels were measured by the frequency of CD107a/MIP-1β in Pol-specific CD8^+^ T cells cocultured with RhT5- or TCyp-expressing cells infected with HIV-1 in the presence or absence of AZT, SmBz-CsA, or CsA. (F) Representative zebra plot showing CD107a/MIP-1β expression in HIV-1 Gag- or Pol-specific CD8^+^ T cells in response to peptide pulse control B cells loaded with cognate peptide. The *P* values were calculated using a Mann-Whitney test. Only significant values are represented (***, *P* < 0.0001; **, *P* < 0.001; *, *P* < 0.01). Experiments shown in panel A and in panels C to E were performed at an MOI of 0.2.

Next, we sought to examine whether TRIM5 antiviral activity was required for direct activation of HIV-1-specific CD8^+^ T-cell responses. To do this, we inhibited TRIM5 activity prior to coculture with HIV-1-specific CD8^+^ T cells and monitored changes in CD8^+^ T-cell activation. Although previous studies have identified HIV-1 mutants with reduced sensitivity to RhT5 and TCyp, these mutants are never fully resistant to TRIM5; in addition, their sensitivity is sequence dependent and varies across cell types (27, 28). Therefore, for our purposes, we decided to use an analogue of CsA, the drug SmBz-CsA (29). We observed that SmBz-CsA acted as an inhibitor of TRIM5 activity by recovering HIV-1 infectivity in cells expressing both RhT5 (from 3% to 13.3%) and TCyp (from 12% to 43%) (Fig. 2A). Moreover, treatment with SmBz-CsA did not affect the functionality of CD8^+^ T cells lacking the immunosuppressive effect of CsA (Fig. 2B). Based on these *in vitro* properties, we used SmBz-CsA to estimate the specific effect of RhT5 and TCyp expression in HIV-1-infected cells on CD8^+^ T-cell activation. To this end, we used two CD8^+^ T cell lines specific for the recognition of two HIV-1 epitopes, one in Gag and one in Pol. We cocultured CD8^+^ T cells with cells expressing RhT5 or TCyp infected with HIV-1 in the presence or absence of SmBz-CsA or CsA and evaluated changes in CD8^+^ T-cell activation based on the expression of CD107a/MIP-1β (Fig. 2C). Consistently, we observed that treatment with SmBz-CsA significantly reduced activation of HIV-1 Gag-specific CD8^+^ T cells in the cocultures (RhT5 versus RhT5 plus SmBz-CsA, *P* = 0.002; TCyp versus TCyp plus SmBz-CsA, *P* = 0.018) (Fig. 2D), indicating a direct effect of TRIM5 expression in CD8^+^ T-cell activation. Meanwhile, a trend was observed for HIV-1 Pol-specific CD8^+^ T cells (Fig. 2E). Moreover, treatment with AZT, resulting in 50% virus inhibition in all of the cell lines used, did not affect CD8^+^ T-cell activation levels. This finding is consistent with the notion that CD8^+^ activation in our experiments results from antigen presentation of the incoming viral particles (Fig. 2D and E). Discrepancies between the Gag- and Pol-specific CD8^+^ T-cell activation levels observed could not be attributed to initial differences in functionality (Fig. 2F), thus suggesting a TRIM5-specific effect.

**FIG 3:**
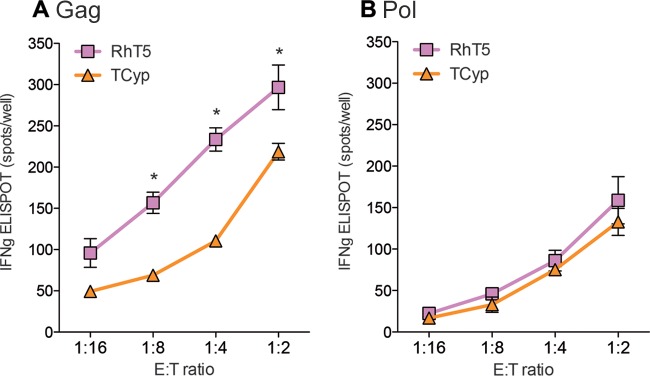
IFN-γ secretion in HIV-1 Gag- and Pol-specific CD8^+^ T cells cocultured with HIV-1-infected cells expressing RhT5 and TCyp. RhT5- and TCyp-expressing cells were infected with HIV-1_GFP_ at an MOI of 0.5 and cocultured with HLA class I-matched HIV-1 Gag- or Pol-specific CD8^+^ T cells at various effector/target (E/T) ratios (1:16, 1:8, 1:4, and 1:2) and incubated overnight. (A) IFN-γ spots per well in Gag-specific CD8^+^ T cells. (B) IFN-γ spots per well in Pol-specific CD8^+^ T cells. Data are from two independent experiments performed in triplicate for panel A and from three independent experiments performed in triplicate for panel B. The *P* values were calculated using a Wilcoxon signed-rank test. Only significant values are shown in the figure (***, *P* < 0.0001; **, *P* < 0.001; *, *P* < 0.01).

We also tested IFN-γ release as an additional marker of CD8^+^ T-cell activation in response to TRIM5 antiretroviral activity. We used an ELISPOT assay to detect IFN-γ owing to the low sensitivity of intracellular cytokine staining under the previous experimental conditions. As shown in Fig. 3A, we observed target-to-effector dose-dependent IFN-γ production of Gag-specific CD8^+^ T cells following coculture with RhT5- and TCyp-expressing cells infected with HIV-1. In addition, we observed differences in IFN-γ production between RhT5 and TCyp variants. Thus, RhT5 showed a higher potency than TCyp for activation of Gag-specific CD8^+^ T cells as measured using IFN-γ production (RhT5 versus TCyp, *P* = 0.0313) (Fig. 3A). In agreement with the previous experiment, the differences observed were specific to CD8^+^ T cells recognizing Gag (Fig. 3B).

**FIG 4:**
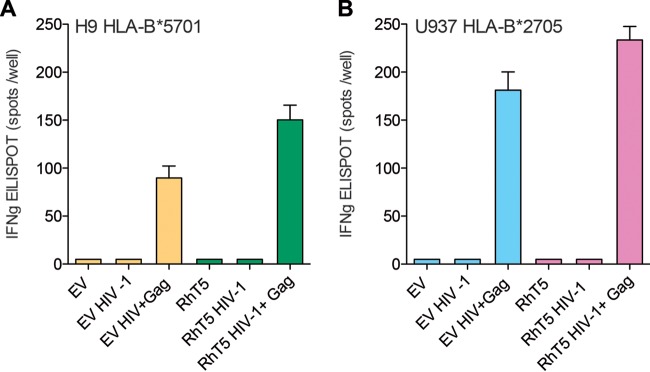
IFN-γ secretion in HIV-1 Gag-specific CD8^+^ T cells cocultured with H9 and U937 HIV-1-infected cells expressing EV and RhT5. H9 HLA-B*5701 and U937 HLA-B*2705 EV- and RhT5-expressing cells were infected with HIV-1_GFP_ at an MOI of 0.5 and cocultured with HLA class I-matched HIV-1 Gag-specific CD8^+^ T cells at an effector/target (E/T) ratio of 1:2 and incubated overnight. (A) IFN-γ spots per well in H9 HLA-B*5701 coculture with HLA-class I-matched Gag-specific CD8^+^ T cells. (B) IFN-γ spots per well in U937 HLA-B*2705 coculture with HLA class I-matched Gag-specific CD8^+^ T cells.

Our findings were further confirmed in H9 HLA-B*5701 EV- or RhT5-expressing cells infected with HIV-1 and coculture with additional HLA-class I-matched Gag-specific CD8^+^ T cells. As shown in Fig. 4A, we observed an increase in IFN-γ production of Gag-specific CD8^+^ T cells in cocultures of RhT5-expressing cells compared to levels in EV-expressing cells. As expected, similar findings were observed in our U937 experimental model (Fig. 4B). Overall, these data support the contribution of RhT5 and TCyp to increased HIV-1-specific CD8^+^ T-cell antiviral activity in response to infection. Moreover, we observed a species-specific effect of the TRIM5 variant and a CD8^+^ T-cell-specific effect on the HIV-1 protein being recognized.

### TRIM5 antiretroviral activity recruits the viral capsid and favors contacts with the cellular proteasome.

**FIG 5:**
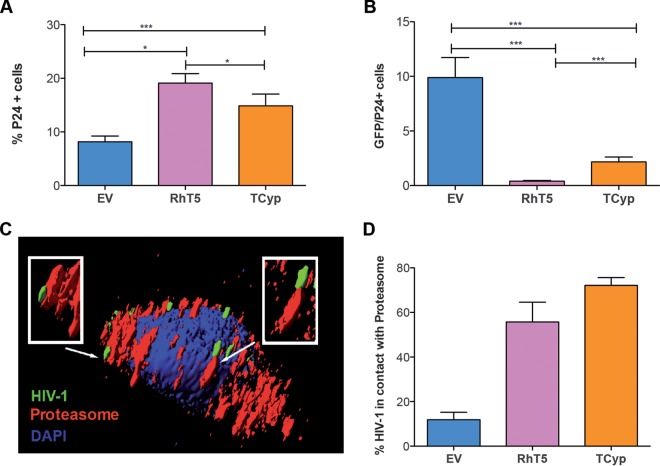
TRIM5 retroviral restriction favors HIV-1 p24^Gag^ recruitment and virus interactions with the cellular proteasome. (A) Intracellular p24^Gag^ levels. (B.) Ratio of GFP/p24-positive cells in EV-, RhT5-, and TCyp-expressing infected cells. Data are from six independent experiments performed in triplicate at an MOI of 0.2. The *P* values were calculated using a Mann-Whitney test. Only significant values are shown in the figure (***, *P* < 0.0001; **, *P* < 0.001; *, *P* < 0.01). (C) Representative image of HIV-1 proteasome interactions in RhT5-expressing cells. The virus NL43-VprGFP is shown in green, the proteasome is shown in red, and the cellular nucleus is shown in blue (DAPI). White arrows and boxed areas show HIV-1 contacts with the cellular proteasome. (D) Number of HIV-1 proteasome contacts in TRIM5-expressing cells. Data represent the number of contacts in five independent cells per condition.

The activation of CD8^+^ T cells relies on efficient antigen presentation through the class I processing pathway (30), which ultimately presents preprocessed viral epitopes on the surface of HLA class I molecules. The association between the level of antigen and the magnitude of specific CD8^+^ T-cell responses (31) suggests a role for TRIM5 expression in delivering viral antigens for HLA class I presentation that will translate into enhanced CD8^+^ T-cell activation. To gain further insights into the mechanism that promotes CD8^+^ T-cell activation in this system, we measured intracellular capsid levels and quantified HIV-1 contacts with the proteasome as an indicator of protein recruitment and degradation for HLA class I presentation. We measured capsid levels by intracellular p24 staining in the three different lines and found an accumulation of p24 in RhT5- and TCyp-expressing cells infected with HIV-1 compared with the level in EV-expressing control cells (EV versus RhT5, *P* < 0.0001; EV versus TCyp, *P* = 0.024) (Fig. 5A) and a reduction in the ratio of GFP/p24 cells in RhT5- and TCyp-expressing cells (EV versus RhT5, *P* < 0.0001; EV versus TCyp, *P* < 0.0001) (Fig. 5B). These data suggest an association between retroviral restriction and accumulation of the viral capsid.

**FIG 6:**
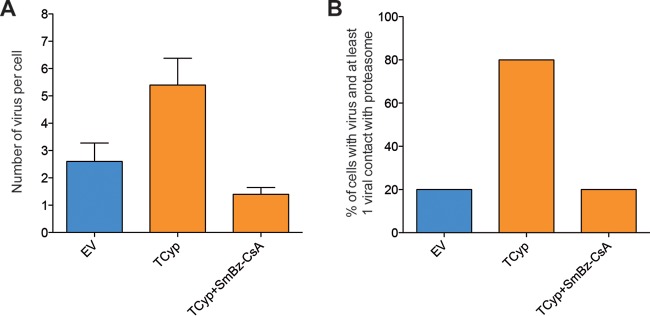
HIV-1 TRIM5-mediated restriction contributes to viral particle recruitment to the proteasome. (A) Number of virus particles per cell. (B) Frequency of cells with viruses with at least a viral contact with the proteasome. Data represent the number of virus/contacts in five independent cells per condition.

We next quantified HIV-1 contacts with the cellular proteasome in TRIM5-expressing cells as indirect measurements of peptide availability for HLA class I loading. We incubated EV-, RhT5-, and TCyp-expressing cells with GFP-Vpr-labeled HIV-1 and stained the cells for the 19S proteasome subunit in DAPI mounting medium (Fig. 5C). We used confocal microscopy to quantify HIV-1 contacts with cellular proteasomes (see Movies S1 and S2 in the supplemental material) and observed an increase in the number of HIV-1 contacts with the cellular proteasome in the presence of RhT5 and TCyp variant expression (median RhT5 value, 56.25%; median TCyp value, 70.18%) compared with EV (9.9%) (Fig. 5D). This observation is consistent with findings from previous reports (32). In addition, the number of virus-proteasome contacts per cell decreased after treatment of TCyp-expressing cells with SmBz-CsA, suggesting a direct role of TRIM5 in virus-proteasome interactions (Fig. 6).

Taken, together these data demonstrate that TRIM5 variants enhance CD8^+^ T-cell activation and that such an increase is accompanied by capsid and proteasome recruitment in HIV-1-infected cells expressing TRIM5 and suggest a direct role for TRIM5 antiviral activity in virus-proteasome interactions.

## DISCUSSION

Devising ways to activate both innate restriction factors and effective CD8^+^ T-cell responses may provide the basis for novel therapeutic strategies that can contribute to HIV-1 control during acute infection (33). Here, we identified a novel role for two nonhuman TRIM5 variants, RhT5 and TCyp, both of which are potent restrictors of HIV-1 and inducers of HIV-1-specific CD8^+^ T-cell responses. We demonstrated that RhT5 and TCyp proteins are able to link innate retroviral restriction and CD8^+^ T-cell antiviral activity to improve cellular protection against retrovirus infection.

**FIG 7:**
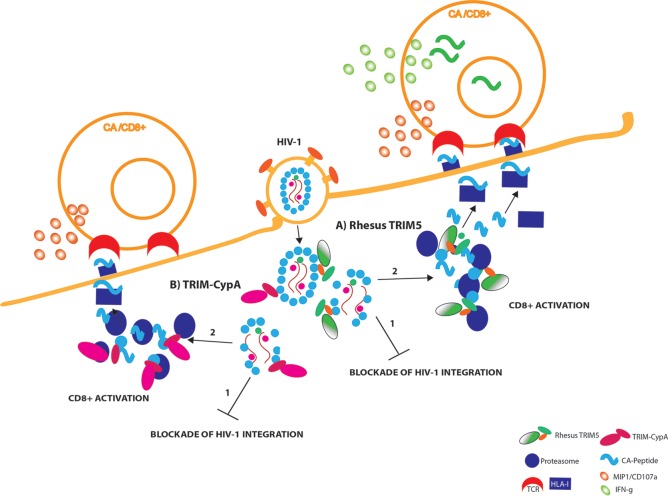
Graphical summary. Once HIV-1 fuses with the cell membrane, the TRIM5 proteins rapidly recognize the incoming viral capsid (CA). (A) Retroviral capsid recognition by rhesus TRIM5 (RhT5) is translated into the following: premature uncoating that will prevent integration of HIV-1 (1) and proteasome engagement by RhT5 and the augmentation of production viral peptides (preferentially for Gag-derived peptides, e.g., capsid) ready to enter the HLA-class I presentation pathway that will enable HIV-1 epitope recognition and CD8^+^ T-cell activation (2). Activation of capsid-specific CD8^+^ T-cell activation will lead to rapid degranulation (CD107a/MIP-1β) and secretion of IFN-γ upon antigen presentation.(B) Similarly, retroviral capsid recognition by TRIM-CypA (TCyp) will lead to following processes: premature viral uncoating that will block integration of HIV-1 (1) and TCyp proteasome recruitment and particle association that will contribute to the generation of viral peptides (preferential capsid peptides) that will enter the class I presentation pathway, thus enabling HIV-1 epitope recognition and early degranulation of CD8^+^ T cells upon recognition of HIV-1-infected cells, albeit to a lesser extent than RhT5 (2). TCR, T-cell receptor.

We envisaged the following model (Fig. 7). The recognition of incoming viral particles by RhT5 and TCyp variants enables at least two antiviral activities: (i) capsid recognition-dependent blockade of HIV-1 integration through the premature uncoating of the viral particles and (ii) activation of CD8^+^ T cells through the recruitment of the viral particles to the cellular proteasome. These interactions lead to increased peptide availability and efficient peptide HLA class I loading and trafficking for antigen presentation by the incoming viral particles. Antigen presentation is more abundant for capsid peptides and RhT5-expressing infected cells, where these interactions will lead to production of IFN-γ (Fig. 7A) and CD107a/MIP-1β in HIV-1-specific CD8^+^ T cells, than for TCyp-expressing infected cells (Fig. 7B).

Although previous studies have evaluated the function of restriction factors in adaptive immunity, their role in antigenicity varied depending on the restriction factor studied and cell type analyzed. APOBEC3G expression is able to promote CD8^+^ T-cell responses, thus favoring peptide availability for proteasome degradation through the generation of misfolded or truncated proteins (19). The presence of SAMHD1 has a negative impact on antigen presentation and CD8^+^ T-cell activation in monocyte-derived dendritic cells (18). Thus, only the restriction factors that promote CD8^+^ T-cell activation in the context of potent retroviral restriction would favor sustained viral control in this way. Together with APOBEC3G, TRIM5 has one of the strongest positive selection signals in the human genome, thereby underscoring its antiviral potency (22). However, human TRIM5 does not restrict HIV-1 owing to evolutionary adaptation of the capsid (34), whereas the primate TRIM5 orthologs RhT5 and TCyp do. Compared with other restriction factors, TRIM5 has a particular advantage as it functions as a rapid viral sensor of incoming HIV-1 particles and therefore likely contributes to prevent seeding of the viral reservoir.

Our data demonstrate a species-specific effect of TRIM5 variants as enhancers of the antigenicity of HIV-1-specific CD8^+^ T-cell responses in infected cells. Thus, RhT5 expression in HIV-1-infected cells induced higher levels of CD8^+^ T-cell activation than that in TCyp-expressing cells. The differences observed may account for divergences in TRIM5 proteins between species. Although the reasons for our findings are not fully understood, we can hypothesize that differences in protein stability (35), capsid recruitment, and affinity for proteasome interactions may account for the differential amounts of antigen being presented and the differences in CD8^+^ T-cell activation profiles.

Along with the species-specific effect on activation, our data revealed an effect on CD8^+^ T-cell viral protein specificity in response to TRIM5 restriction. Thus, CD8^+^ T cells targeting a viral capsid epitope display higher levels of activation in response to RhT5- and TCyp-expressing cells infected with HIV-1 than in Pol-specific CD8^+^ T cells. It is not clear why Pol epitopes, for example, those derived from protease, integrase, or reverse transcriptase, are not more effectively presented in RhT5- and TCyp-expressing cells. We hypothesize that this may due to the smaller amounts of protein in the viral particle than those observed in Gag. However, it may also be due to preferred degradation and presentation of Gag-derived peptides because it is Gag-derived capsid protein that is actually bound by TRIM5/TCyp. Intrinsic epitope properties may also play a role (24, 35). Further studies will help to clarify whether our findings extend to other HIV-1 CD8^+^ T-cell responses with different epitope specificities and/or avidity.

Previous studies suggested that HLA-B*27 can present peptides independently of proteasomes and TAP (transporter associated with antigen processing) (36). However, epitope abundance predicted by proteasomal digestion correlated with the hierarchies of CD8^+^ T-cell responses in HLA-B*27 HIV-1-infected individuals (37). These data suggest that the contribution of proteasome-negative, TAP-negative antigen presentation is of minor importance in fully functional cells, where proteasome-dependent pathways outcompete alternative antigen presentation pathways (38).

The precise mechanistic details of this mechanism of CD8^+^ T-cell-mediated activation of TRIM5 should be investigated in further detail. However, we hypothesized that they may be dependent on the TRIM5 E3 ligase activity that promotes the generation of polyubiquitinated chains of TRIM5 proteins, leading to their subsequent recruitment to the cellular proteasome. The role of polyubiquitination of TRIM proteins was recently described as a mechanism to synergize innate signaling and virus degradation in TRIM21, TRIM5, and TRIM25 (7, 10, 39).

We establish a new mechanism of action for nonhuman TRIM5 proteins in HIV-1 specific CD8^+^ T-cell activation.

Our study is limited to the use of immortalized cell lines, and further studies are ongoing to evaluate the contribution of TRIM5 restriction to CD8^+^ T-cell antigen presentation in primary CD4^+^ lymphocytes. Although human TRIM5 is not thought to strongly restrict HIV-1 infection in humans, we hypothesized that in certain cases, for example, under selective pressure to escape Gag-directed CD8^+^ T-cell responses (40), HIV-1 variants sensitive to human TRIM5 may arise. In this case, we expect human TRIM5 to contribute to T-cell-mediated killing of infected cells, as described above.

The function of TRIM5 proteins as restriction factors and pattern recognition receptors (10), together with the function described here as enhancers of HIV-1 CD8^+^ T-cell recognition, make them good candidates for novel therapeutic strategies against HIV-1. Such therapies could target the generation of novel gene therapy vectors, where promising results have already been obtained (41, 42), or small molecules that mimic the function of TRIM5 proteins. In this context, TRIM5-based gene therapy vectors will have an additional protective immune function through induction of antiviral CD8^+^ T-cell responses.

Our findings offer new insights into the interplay between HIV-1, TRIM5 retroviral restriction, and adaptive cellular immunity that should be explored in detail to develop innovative strategies that can be widely applied to control retroviruses.

## Supplementary Material

Supplemental material
