# Supplementary material for: Nonhuman TRIM5 Variants Enhance Recognition of HIV-1-Infected Cells by CD8+ T Cells
Source: J Virol. 2016 Sep 12;90(19):8552–62. doi: 10.1128/JVI.00819-16 (PMC5021395; doi:10.1128/JVI.00819-16)
Supplement: Supplemental material [file supp_90_19_8552__index.html]

Nonhuman TRIM5 Variants Enhance Recognition of HIV-1-Infected Cells by CD8+ T Cells — Supplemental material 

# Nonhuman TRIM5 Variants Enhance Recognition of HIV-1-Infected Cells by CD8+ T Cells

## Supplemental material

- Supplemental file 1 -

  Movie S1 (Virus proteasome contacts in EV cells.)

  MOV, 10M
- Supplemental file 2 -

  Movie S2 (Virus proteasome contacts in RhT5 cells.)

  MOV, 12M
